# Supplementary material for: The accessory type III secretion system effectors collectively shape intestinal inflammatory infection outcomes
Source: Gut Microbes. 2025 Jul 2;17(1):2526134. doi: 10.1080/19490976.2025.2526134 (PMC12233879; doi:10.1080/19490976.2025.2526134)
Supplement: Table S1.docx [file KGMI_A_2526134_SM8052.docx]

**Table S1: Function and activity (during infection) of core CR accessory effectors.**

*In vivo roles are described in* ***bold****.*

| **Effectors** | **Structure and function** | **Role during infection** | **Refs.** |  |
| --- | --- | --- | --- | --- |
| Map | WxxxE GEF that targets Cdc42, EBP50, localized to the mitochondria | Disrupts mitochondrial membrane potential, causes Ca^2+^ efflux, disruption of mitochondrial morphology and function, caspase activation, and host cell apoptosis; mimics RhoGEF activity, cytoskeleton remodeling (transient filopodia formation), **disrupts tight junction** | ^1-5^ |  |
| NleD1 & NleD2 | Zinc Metalloprotease, directly cleaves and inactivates JNK, p38 | MAPK signaling inhibition, blocks the transcription of proapoptotic & proinflammatory genes initiated by AP-1 transcript | ^6-9^ |  |
|  |  |  |  |  |
| NleH | Ser/Thr Kinase, binds BI-1 and RPS3; *in vivo* NleH1 can suppress ERK1/2 and p38, along with NF-kβ signaling and NleH2 only suppresses caspase-3 and p38, phosphorylates Esp8 | **Inhibit inflammatory responses through targeting the NF-kβ pathway & its nuclear translocation**, **inhibits death signaling**, might alter cytoskeletal composition of AE lesions during EPEC infection | ^10-15^ |  |
| NleF | Caspase inhibitor (caspase-4, 8 & 9), NleF_CR_ inhibits caspase-11 | Inhibits intrinsic and extrinsic apoptotic and pyroptotic cell death. **Inhibits IL-18 secretion** | ^16,17^ |  |
| EspJ | Amidation and ADP ribosylation of host non-receptor tyrosine kinases | Inhibits phagocytosis | ^18,19^ |  |
| NleG1 & NleG7 | E3 Ubiquitin ligase | NleG7 contributes to bacterial survival during enteric infection, and NleG1 promotes the expression of diarrheal symptoms | ^20,21^ |  |
| EspV |  | Actin remodeling and morphological changes in mammalian cells | ^22^ |  |
| EspK |  | Related to anti-inflammatory effectors GogB (Salmonella, GogB inhibits NF-kβ signaling) and YopM (Yersinia, YopM blocks YopE-induced Pyrin inflammasome activation) | ^23^ |  |
| NleN, NleK |  | Unknown | ^24,25^ |  |

**References**

1 Singh, A. P. *et al.* Enteropathogenic E. coli effectors EspF and Map independently disrupt tight junctions through distinct mechanisms involving transcriptional and post-transcriptional regulation. *Sci Rep* **8**, 3719 (2018). <https://doi.org/10.1038/s41598-018-22017-1>

2 Ma, C. *et al.* Citrobacter rodentium infection causes both mitochondrial dysfunction and intestinal epithelial barrier disruption in vivo: role of mitochondrial associated protein (Map). *Cell Microbiol* **8**, 1669-1686 (2006). <https://doi.org/10.1111/j.1462-5822.2006.00741.x>

3 Dean, P. & Kenny, B. Intestinal barrier dysfunction by enteropathogenic Escherichia coli is mediated by two effector molecules and a bacterial surface protein. *Mol Microbiol* **54**, 665-675 (2004). <https://doi.org/10.1111/j.1365-2958.2004.04308.x>

4 Kenny, B. & Jepson, M. Targeting of an enteropathogenic Escherichia coli (EPEC) effector protein to host mitochondria. *Cell Microbiol* **2**, 579-590 (2000). <https://doi.org/10.1046/j.1462-5822.2000.00082.x>

5 Kenny, B. *et al.* Co-ordinate regulation of distinct host cell signalling pathways by multifunctional enteropathogenic Escherichia coli effector molecules. *Mol Microbiol* **44**, 1095-1107 (2002). <https://doi.org/10.1046/j.1365-2958.2002.02952.x>

6 Alto, N. M. *et al.* Identification of a bacterial type III effector family with G protein mimicry functions. *Cell* **124**, 133-145 (2006). <https://doi.org/10.1016/j.cell.2005.10.031>

7 Creuzburg, K. *et al.* The Type III Effector NleD from Enteropathogenic Escherichia coli Differentiates between Host Substrates p38 and JNK. *Infect Immun* **85** (2017). <https://doi.org/10.1128/IAI.00620-16>

8 Gur-Arie, L., Eitan-Wexler, M., Weinberger, N., Rosenshine, I. & Livnah, O. The bacterial metalloprotease NleD selectively cleaves mitogen-activated protein kinases that have high flexibility in their activation loop. *J Biol Chem* **295**, 9409-9420 (2020). <https://doi.org/10.1074/jbc.RA120.013590>

9 Baruch, K. *et al.* Metalloprotease type III effectors that specifically cleave JNK and NF-kappaB. *EMBO J* **30**, 221-231 (2011). <https://doi.org/10.1038/emboj.2010.297>

10 Kralicek, S. E., Nguyen, M., Rhee, K. J., Tapia, R. & Hecht, G. EPEC NleH1 is significantly more effective in reversing colitis and reducing mortality than NleH2 via differential effects on host signaling pathways. *Lab Invest* **98**, 477-488 (2018). <https://doi.org/10.1038/s41374-017-0016-1>

11 Hemrajani, C. *et al.* NleH effectors interact with Bax inhibitor-1 to block apoptosis during enteropathogenic Escherichia coli infection. *Proc Natl Acad Sci U S A* **107**, 3129-3134 (2010). <https://doi.org/10.1073/pnas.0911609106>

12 Hemrajani, C. *et al.* Role of NleH, a type III secreted effector from attaching and effacing pathogens, in colonization of the bovine, ovine, and murine gut. *Infect Immun* **76**, 4804-4813 (2008). <https://doi.org/10.1128/IAI.00742-08>

13 Royan, S. V. *et al.* Enteropathogenic E. coli non-LEE encoded effectors NleH1 and NleH2 attenuate NF-kappaB activation. *Mol Microbiol* **78**, 1232-1245 (2010). <https://doi.org/10.1111/j.1365-2958.2010.07400.x>

14 Gao, X. *et al.* Bacterial effector binding to ribosomal protein s3 subverts NF-kappaB function. *PLoS Pathog* **5**, e1000708 (2009). <https://doi.org/10.1371/journal.ppat.1000708>

15 Pollock, G. L. *et al.* Targeting of microvillus protein Eps8 by the NleH effector kinases from enteropathogenic E. coli. *Proc Natl Acad Sci U S A* **119**, e2204332119 (2022). <https://doi.org/10.1073/pnas.2204332119>

16 Blasche, S. *et al.* The E. coli effector protein NleF is a caspase inhibitor. *PLoS One* **8**, e58937 (2013). <https://doi.org/10.1371/journal.pone.0058937>

17 Pallett, M. A. *et al.* Bacterial virulence factor inhibits caspase-4/11 activation in intestinal epithelial cells. *Mucosal Immunol* **10**, 602-612 (2017). <https://doi.org/10.1038/mi.2016.77>

18 Young, J. C. *et al.* The Escherichia coli effector EspJ blocks Src kinase activity via amidation and ADP ribosylation. *Nat Commun* **5**, 5887 (2014). <https://doi.org/10.1038/ncomms6887>

19 Pollard, D. J. *et al.* Broad-Spectrum Regulation of Nonreceptor Tyrosine Kinases by the Bacterial ADP-Ribosyltransferase EspJ. *mBio* **9** (2018). <https://doi.org/10.1128/mBio.00170-18>

20 Popov, G. *et al.* Distinct Molecular Features of NleG Type 3 Secreted Effectors Allow for Different Roles during Citrobacter rodentium Infection in Mice. *Infect Immun* **91**, e0050522 (2023). <https://doi.org/10.1128/iai.00505-22>

21 Wu, B. *et al.* NleG Type 3 effectors from enterohaemorrhagic Escherichia coli are U-Box E3 ubiquitin ligases. *PLoS Pathog* **6**, e1000960 (2010). <https://doi.org/10.1371/journal.ppat.1000960>

22 Arbeloa, A., Oates, C. V., Marches, O., Hartland, E. L. & Frankel, G. Enteropathogenic and enterohemorrhagic Escherichia coli type III secretion effector EspV induces radical morphological changes in eukaryotic cells. *Infect Immun* **79**, 1067-1076 (2011). <https://doi.org/10.1128/IAI.01003-10>

23 Vlisidou, I. *et al.* Identification and characterization of EspK, a type III secreted effector protein of enterohaemorrhagic Escherichia coli O157:H7. *FEMS Microbiol Lett* **263**, 32-40 (2006). <https://doi.org/10.1111/j.1574-6968.2006.00410.x>

24 Ruano-Gallego, D. *et al.* Type III secretion system effectors form robust and flexible intracellular virulence networks. *Science* **371** (2021). <https://doi.org/10.1126/science.abc9531>

25 Deng, W. *et al.* A comprehensive proteomic analysis of the type III secretome of Citrobacter rodentium. *J Biol Chem* **285**, 6790-6800 (2010). <https://doi.org/10.1074/jbc.M109.086603>
