# Supplementary material for: The accessory type III secretion system effectors collectively shape intestinal inflammatory infection outcomes
Source: Gut Microbes. 2025 Jul 2;17(1):2526134. doi: 10.1080/19490976.2025.2526134 (PMC12233879; doi:10.1080/19490976.2025.2526134)

Supplementary Figure 1

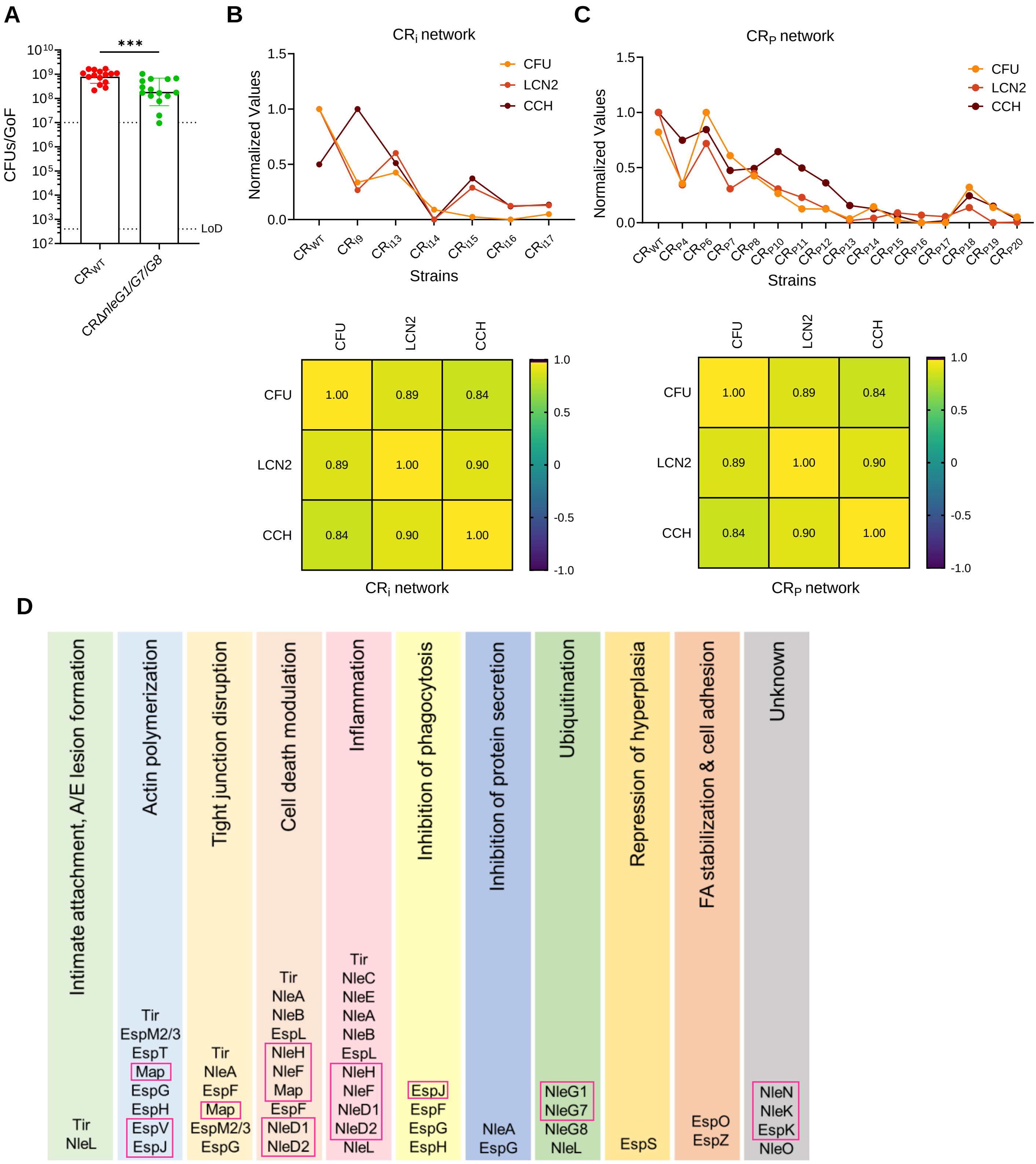

Supplementary Figure 2

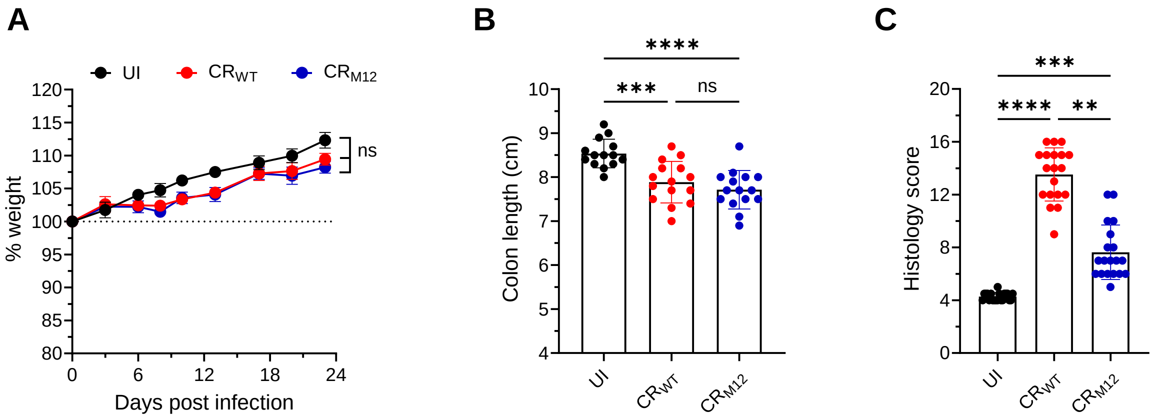

Supplementary Figure 3

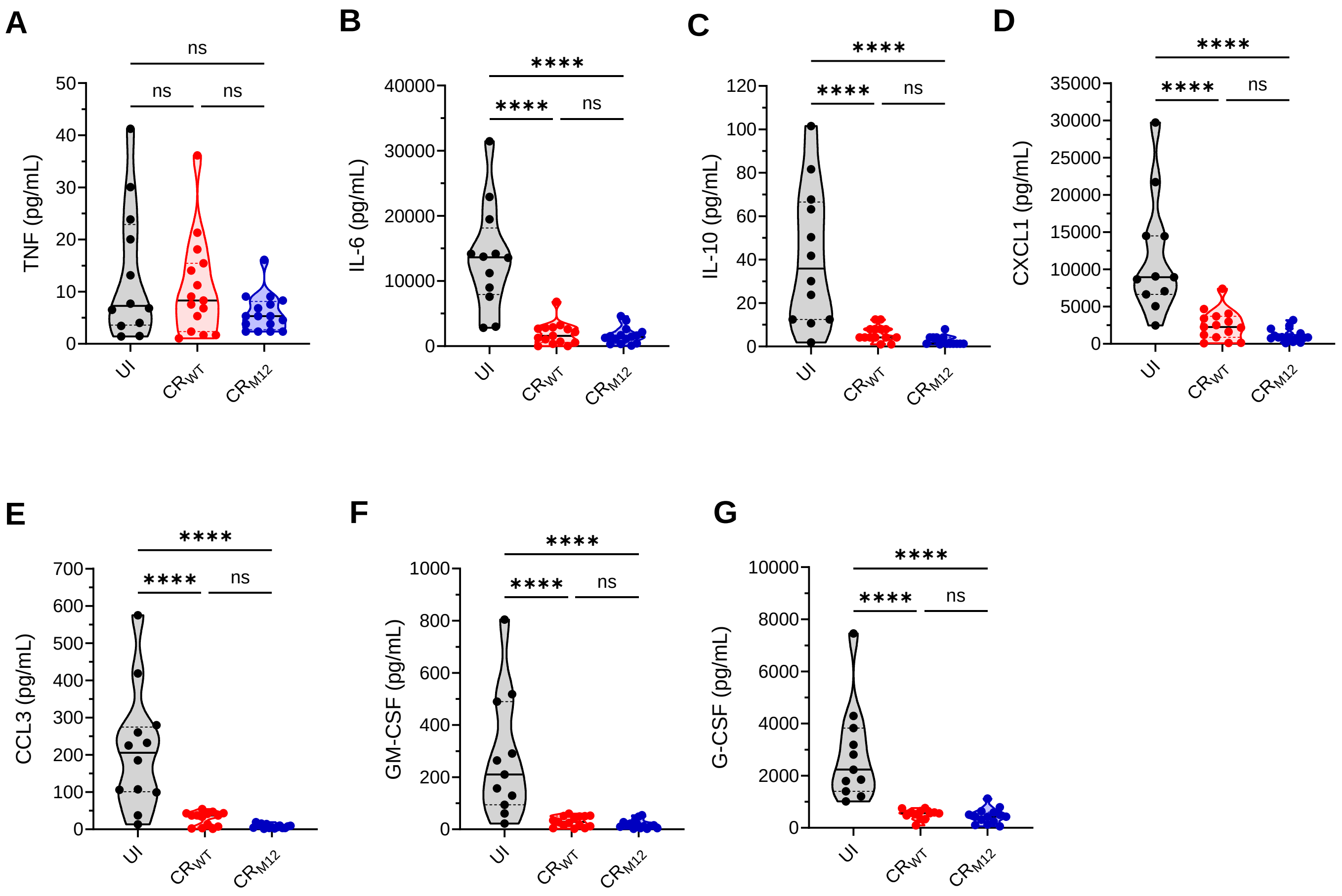

Supplementary Figure 4

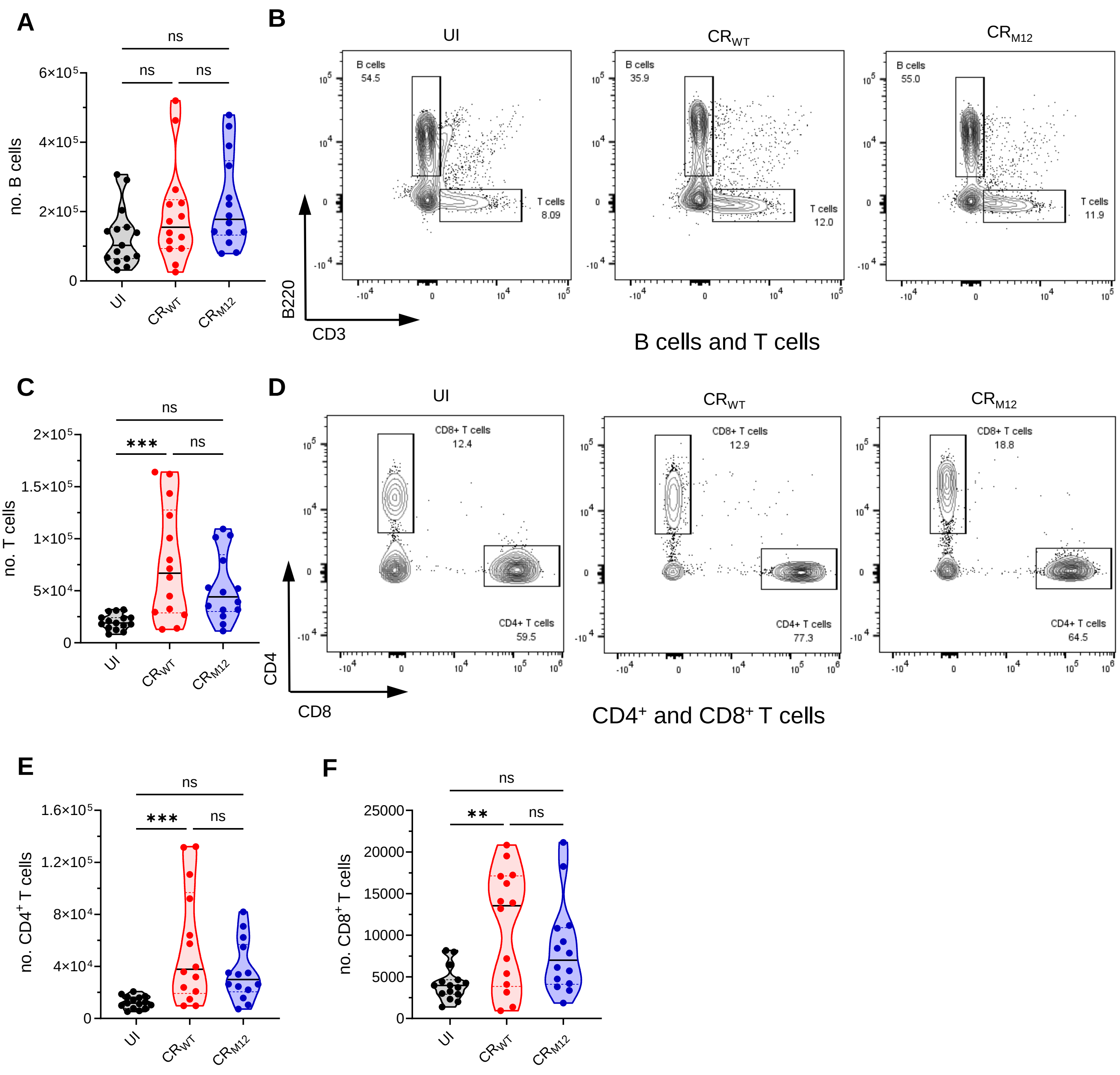

Supplementary Figure 5

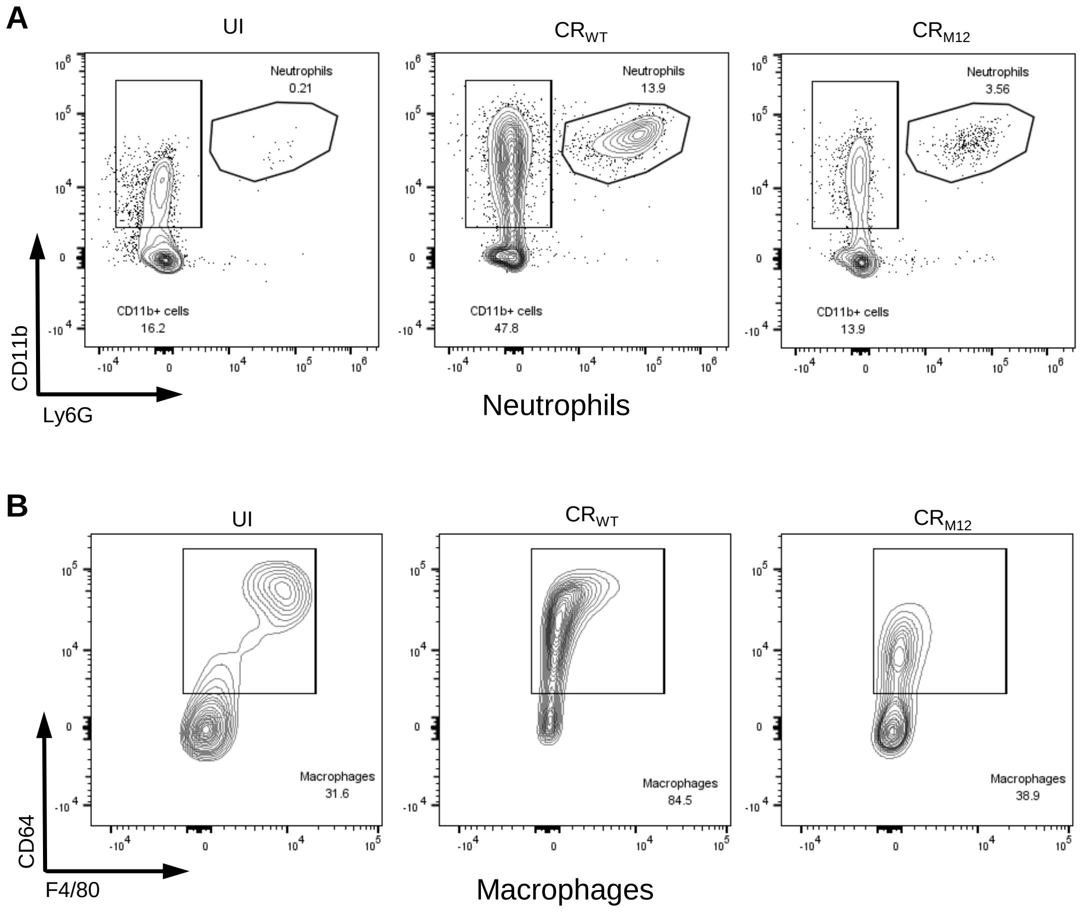

Supplementary Figure 6

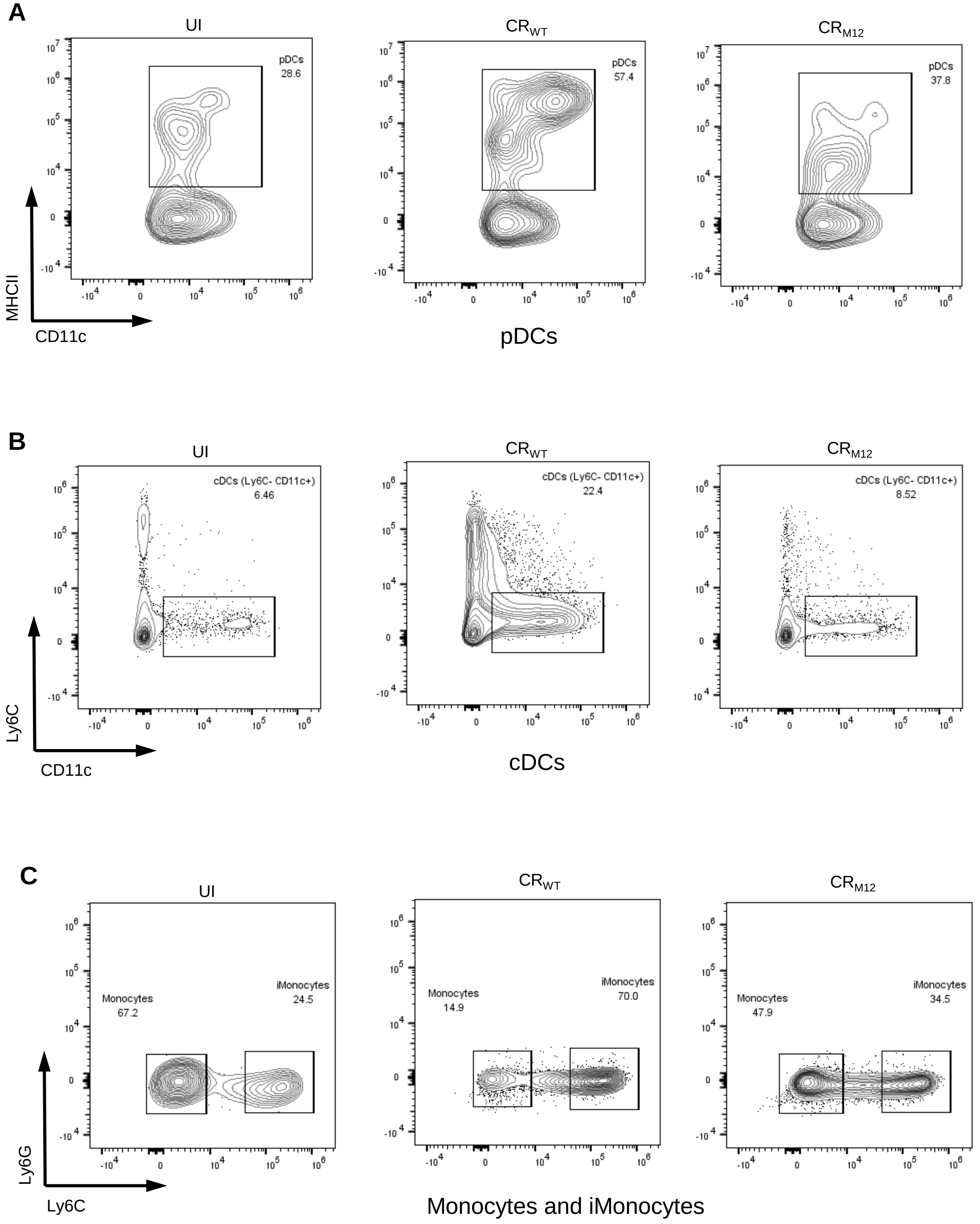

Supplementary Figure 7

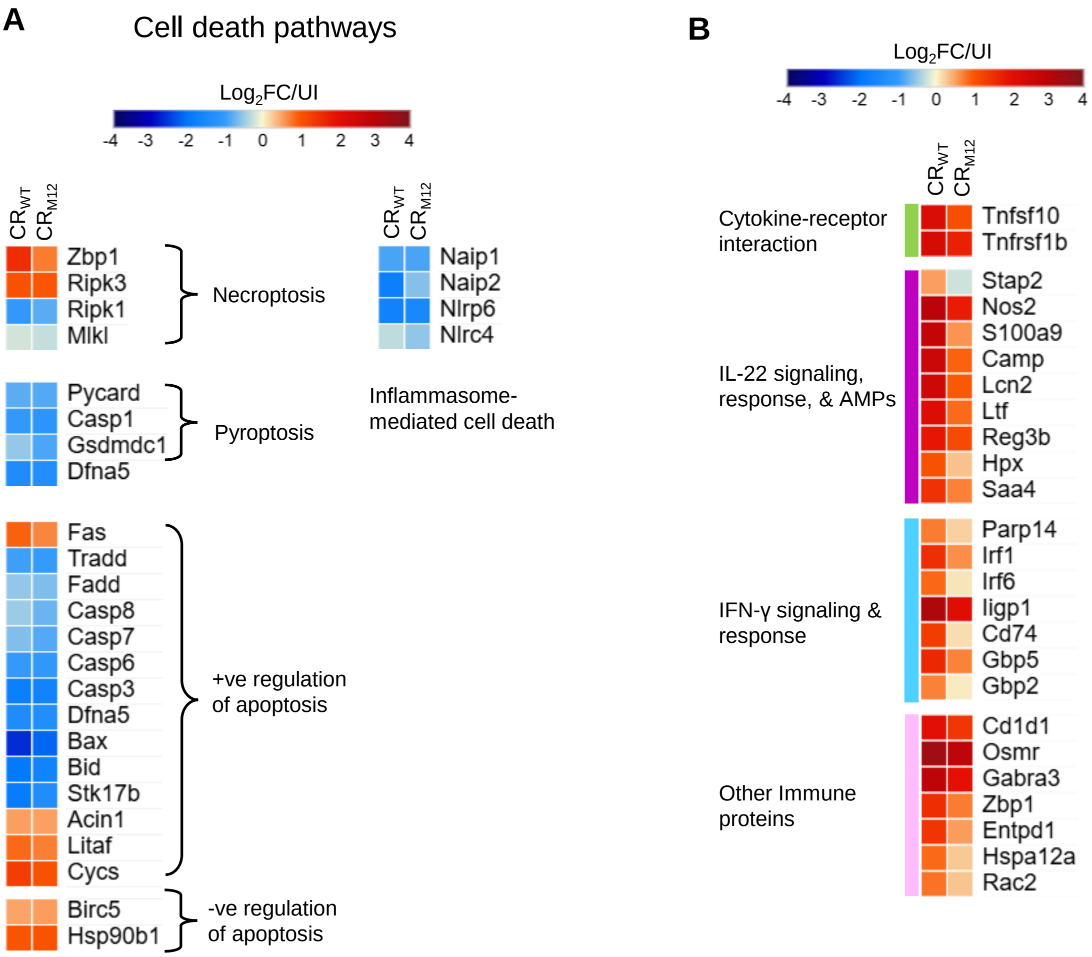

Supplementary Figure 8

A

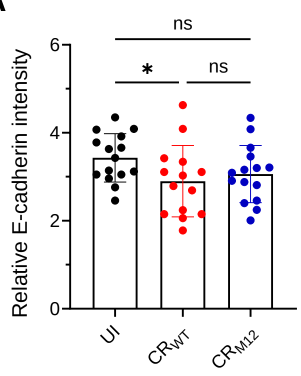

Supplementary Figure 9

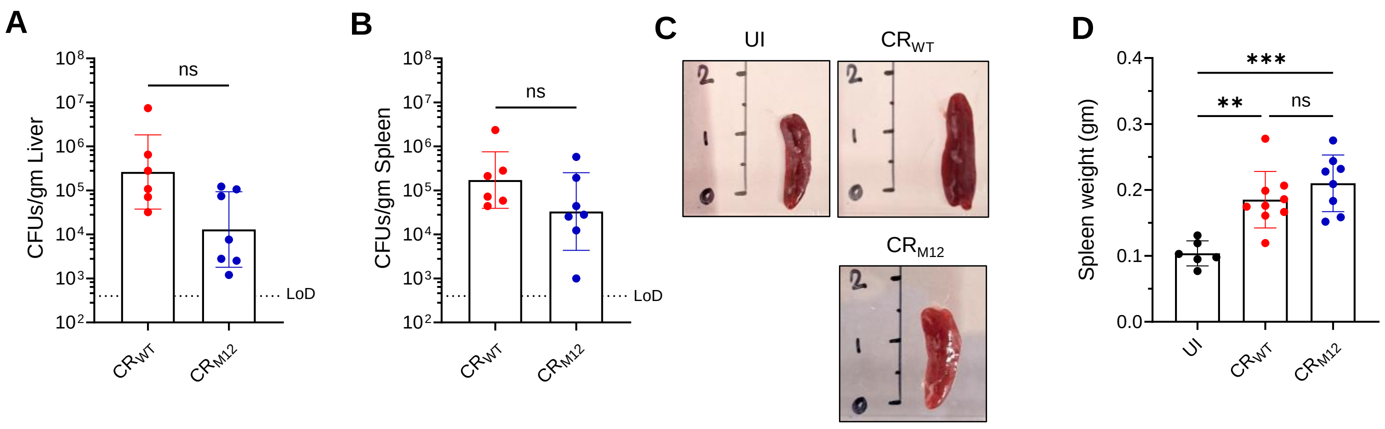

Supplementary Figure 10

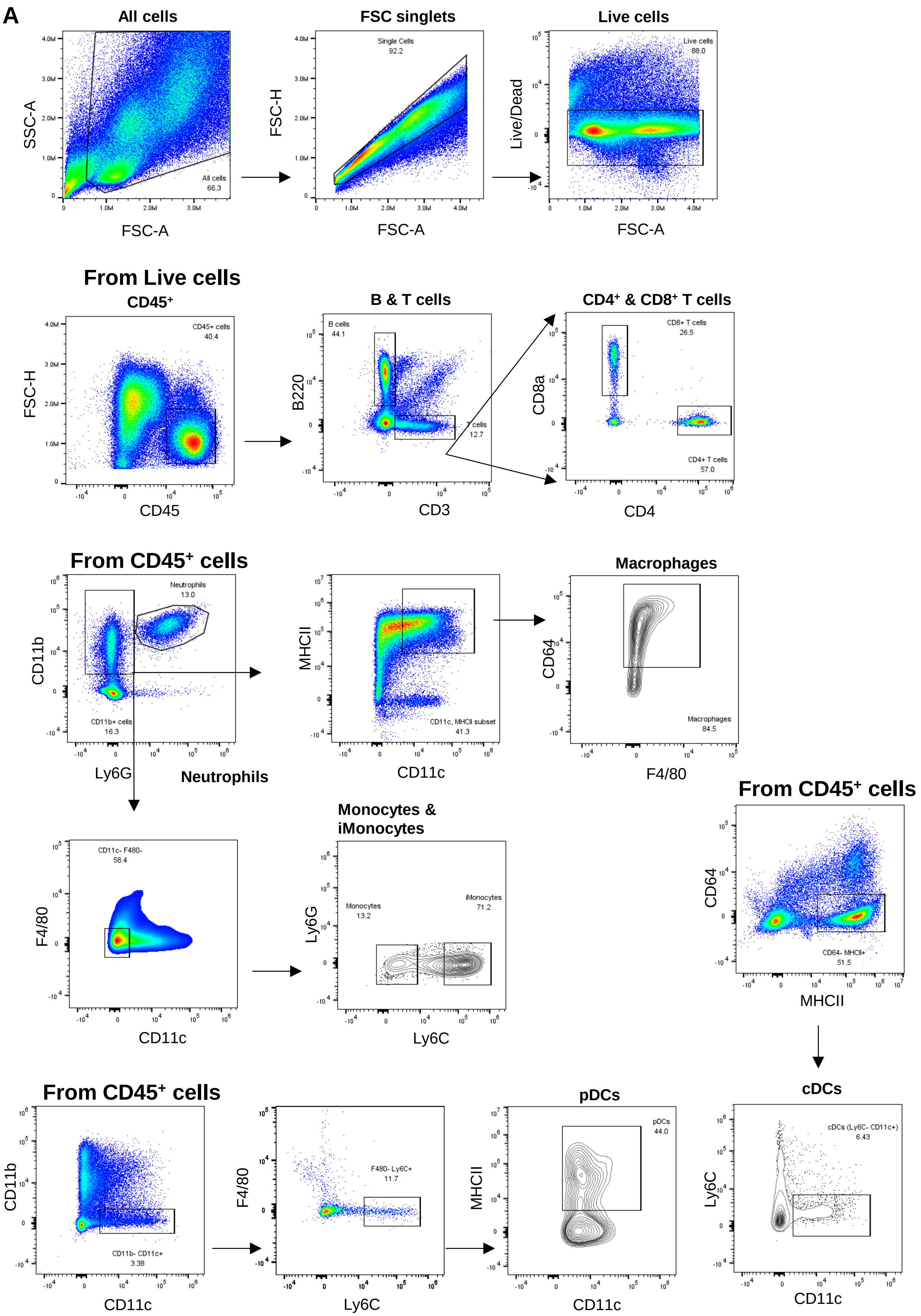

Supplement: Supplementary figures_.pdf [file KGMI_A_2526134_SM8051.pdf]
